# Stress Distribution on Maxillary Canines Following Restoration With Different Dimensions of Metal and Fiber Posts: A Finite Element Study

**DOI:** 10.7759/cureus.53266

**Published:** 2024-01-30

**Authors:** Mahesh Mohan, Lubna Mohammad, Nasarudheen Cholayil, Saumya Vats, Mohammed Salman Kuttikkodan, John Kodumbilayiparambil Anto

**Affiliations:** 1 Department of Conservative Dentistry and Endodontics, Institute of Dental Studies & Technologies, Modinagar, Ghaziabad, IND; 2 Department of Endodontics, Danat Al Sahraa Medical Company, Jubail, SAU; 3 Department of Conservative Dentistry and Endodontics, Sree Anjaneya Institute of Dental Sciences, Kozhikode, IND; 4 Department of Conservative Dentistry and Endodontics, Educare Institute of Dental Science, Malappuram, IND

**Keywords:** titanium post, stress distribution, glass fiber post, finite-element analysis, composite core

## Abstract

Introduction

In recent times, finite element analysis (FEA) in the field of dentistry has been employed to assess the mechanical properties of biological materials and tissues, which are difficult to quantify directly within a living organism. Only a limited number of studies have examined the impact of post diameter and length on how stress is dispersed in a maxillary canine tooth. Hence, this in vitro investigation was conducted to analyze the distribution of stress in a maxillary canine tooth that was replaced using metal and fiber posts with different diameters (1.5 mm and 1.8 mm) and lengths (11 mm and 15 mm), applying FEA.

Materials and methods

A FEA study was performed and all models were grouped as follows: Models 1 and 5 were made of titanium (Ti) and glass fiber posts, respectively, with a diameter of 1.5 mm and a length of 15 mm with composite core and all-ceramic crown; Models 2 and 6 were made of Ti and glass fiber posts, respectively, with a diameter of 1.5 mm and a length of 11 mm with composite core and all-ceramic crown; Models 3 and 7 were made of Ti and glass fiber posts, respectively, with a diameter of 1.8 mm and a length of 15 mm with composite core and all-ceramic crown; and Models 4 and 8 were made of Ti and glass fiber posts, respectively, with a diameter of 1.8 mm and a length of 11 mm with composite core and all-ceramic crown. A force of 200 N was exerted on the ceramic crown at an angulation of 45° to the longitudinal axis of the tooth on the palatal surface above the cingulum. The failure was determined by the correlation between a larger von Mises stress estimate and an increased likelihood of failure. The resulting stresses were then contrasted with the highest possible tensile strength of the material.

Results

The study demonstrated that fiber posts with a diameter of 1.8 mm and an average length of 11 mm exhibited reduced stress levels in comparison to Ti posts. The largest stresses were seen at the cervical region of the tooth, regardless of the materials employed. There was no discernible alteration in stress when the length and diameter of the post were modified. The highest stress in the composite core was measured in Ti posts measuring 1.5 mm in diameter and 15 mm in length. The highest level of stress on dentin was noted in cases where a fiber post was used, as opposed to cases where a Ti post was used. The measured stress within the fiber post was insignificant. However, the pressures imparted to the dentin were greater and more uniformly distributed in comparison to the Ti post cases.

Conclusion

It is suggested that a composite resin core be used along with a fiber post that is larger in diameter and smaller in length, within clinical bounds, in order to lessen stress in the radicular tooth, despite the substantial coronal defect. Further clinical trials are required to assess the survival rate of these specific measurements, dimensions, and biomaterials.

## Introduction

Endodontically restored teeth that have had substantial decay of the tooth often need a post to be inserted into the root canals to support a core for the final restoration [1]. Nevertheless, research conducted both in laboratory settings and in living organisms has shown that a post does not provide additional support to teeth that have undergone endodontic treatment, even though it does enhance retention when there is no more than 3-4 mm of vertical clearance or approximately 25-50% of the visible portion of the tooth [2]. Recently, there have been several advancements in the development of fiber posts. Studies have shown that using a combination of fiber posts, resin, and dentin bonding strategies to restore pulpless teeth has resulted in outstanding long-term clinical outcomes [3-5]. Additionally, it offers an underpinning on which a final restoration can be anchored to the tooth [1].

In the molar area, the mean maximal biting force among humans was determined at 911 N, and in the incisal region, at 569 N. The application of forces to dental restorative materials can potentially lead to deformations caused by alterations to dimensions such as length, volume, remaining coronal tooth structure, choice of material, and material characteristics like modulus of elasticity and stress distribution [6]. Nevertheless, these indicators lack precise definitions. Traditionally, roots that are structurally challenged have been repaired using casting posts and cores, which offer the benefits of post rigidity, optimal adaptability, and strong retention. Titanium (Ti) and nickel-chromium (Ni-Cr) are examples of metallic posts. More recent materials, such as ceramics and fiber-reinforced resin, are employed due to their distinctive aesthetic traits and other advantageous characteristics [1]. The posts’ physical qualities directly impact the stress distribution of the existing tooth structure. Therefore, the precise preference for postings remains a challenging decision for professionals.

Almost all post materials necessitate an enlargement in diameter to get desirable physical properties, ensuring adequate resistance against functional and parafunctional stresses while preventing post fracture. Conversely, the space for the post should be cautiously prepared, ensuring that a minimum thickness of 1 mm of the soundproof dentinal wall is left surrounding the post. When larger metal posts are utilized, they cannot form a strong connection with the root structure. This raises the chances of a root fracture during normal usage. Non-metallic posts, by their capacity to connect with dentin, result in the uniform distribution of stress throughout the root, hence enhancing tooth resistance to fracture [7].

Several authors presented recommendations regarding the ideal length of a post. According to Neagley, a post must be at least 8 mm long [8]. A minimum crown post length ratio of 1:1 was proposed. Various methods have been developed for measuring and analyzing stress, including the strain gauge technique, the loading test, and the photoelastic method. Nevertheless, these solutions possess their own drawbacks. These procedures are analogous, two-dimensional, and challenging to replicate. Creating a reliable indicator of stress distribution in root systems has proven challenging when relying exclusively on experimental and clinical observation. The finite element method (FEM) is a mathematical technique that offers a flexible approach to analyzing stress distributions in complicated systems. The benefits of this approach include a closer approximation to natural settings, lower experimentation costs, prevention of damaging testing, high reproducibility and accuracy of outcomes, and time savings. This technique is highly valuable for assessing the mechanical properties of biological materials and human tissues that are difficult to evaluate directly in living organisms [9]. This technique involves representing a physical structure as a collection of a limited number of pieces. A general approximation solution to the original problem is determined [10].

The impact of post length and diameter on roots’ ability to withstand fracture is still up for debate, despite earlier research. Therefore, a three-dimensional (3D) finite element analysis (FEA) was performed on the maxillary canine tooth to assess and contrast the pattern of stress when a load of 200 N is imposed following the insertion of Ti and fiber posts with diameters of 1.5 mm and 1.8 mm and lengths of 15 mm and 11 mm [7], where the load of 200 N was used to simulate the typical biting or chewing forces that a maxillary canine tooth experiences during normal use.

## Materials and methods

This was an FEA study conducted at Royal Dental College, Kerala, India. The construction of the FEM comprised several steps:

Creating a geometric representation of a typical upper canine tooth

**Figure 1:**
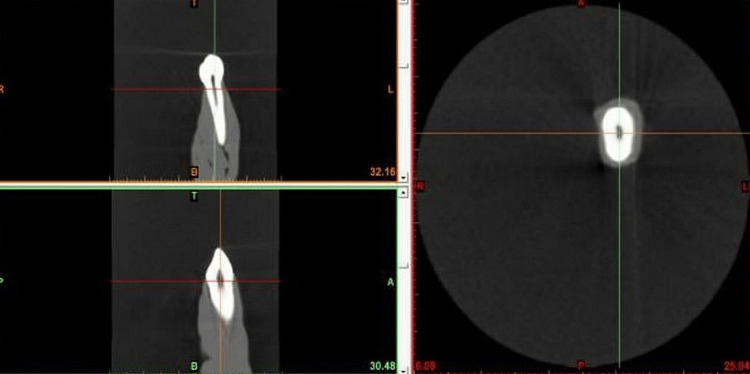
CBCT scans for the FEA CBCT, cone beam computed tomography; FEA, finite element analysis

Modeling is the initial stage of FEA. A mathematical FEA model was developed to analyze sound extracted from human maxillary canines. The preciseness of the model directly impacts the level of accuracy of the outcomes of the analysis. A tooth of approximately 26 mm in length, with a crown height of 10 mm and a root length of 16 mm, was chosen. The tooth had a cone beam computed tomography (CBCT) scan by Carestream Dental LLC (Atlanta, Georgia, United States), 2 mA, 70 kV, and a scan time of 17.5 seconds. The slice interval and slice thickness were both 0.640 mm. Using 3D digital dentofacial diagnostic and imaging technology in Kerala, the various facets of the tooth were observed with precision. The CT scan visuals were acquired in the DICOM format and utilized as input in the Mimics software Version 8.11 (Materialise, Leuven, Belgium). Figure 1 exhibits the utilization of the software for converting the CBCT scan into a 3D geometric framework.

**Figure 2:**
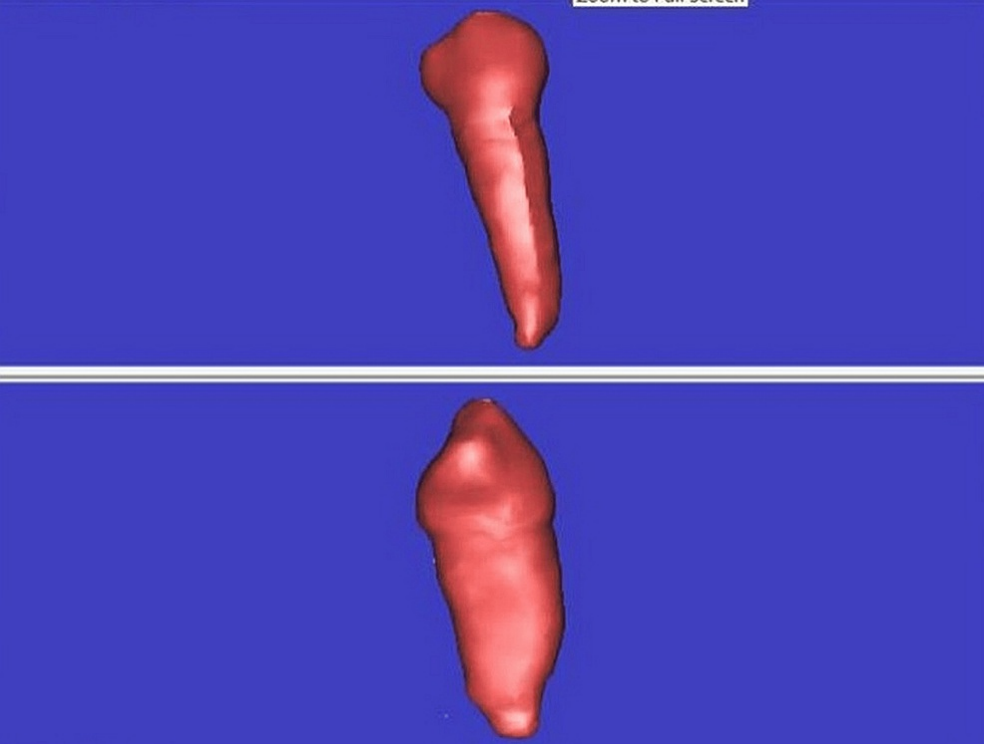
STL image creation in the software STL, stereolithography

Mimics is a software specifically designed for medical purposes that enables the visualization and segmentation of CT and MRI scans. The investigation involved exporting data from the Mimics program, which consisted of cloud data points and lines that are in stereolithography format. This data was then integrated into RapidForm software (Inus Technology, Inc., Seoul, South Korea) to transform the cloud data points into surfaces, including points, lines, and surfaces (Figure 2).

Using a geometric model (GM) to construct a FEM

**Figure 3:**
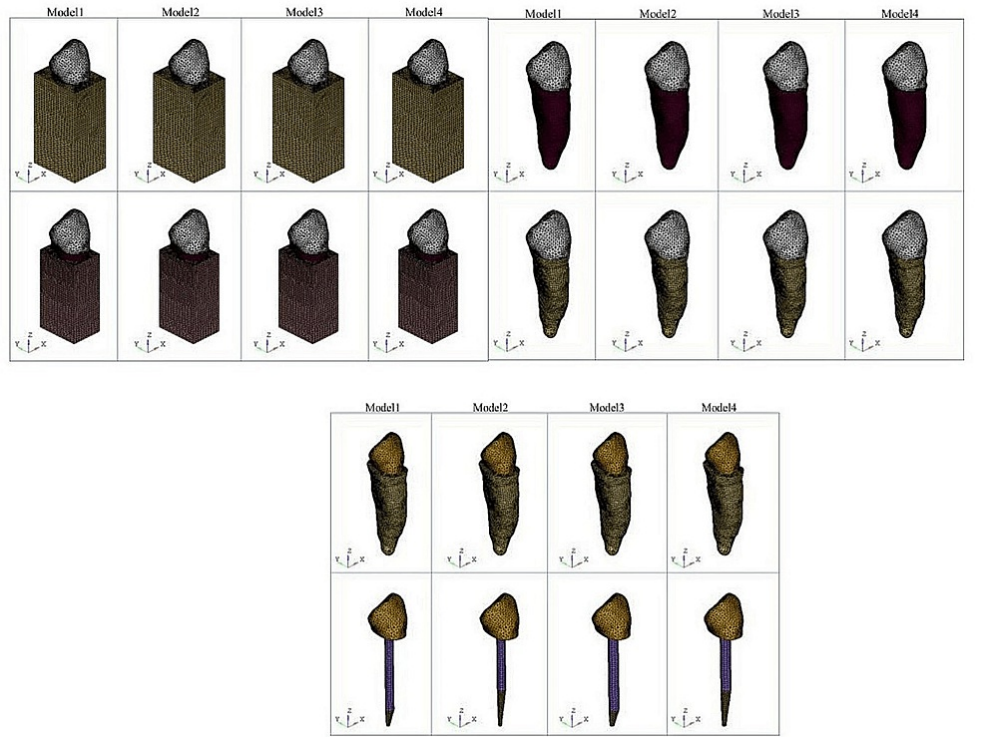
FEMs created for the study FEM, finite element method

The GM was transformed into a FEM employing HYPERMESH software Version 13.0 (Altair, Troy, Michigan, United States). The tooth’s GM was put into the meshing software “Hypermesh.” The Hypermesh program, namely the Altair HyperWorks Version 13.0, was utilized to transform a GM into a FEM. This software offers the benefits of enhancing product performance, automating design procedures, and increasing profitability in a versatile and adaptable setting. The individual components in Hypermesh, such as teeth, periodontal ligament, gutta-percha, post, core, root, and crown, were subsequently divided into smaller elements (meshing) and put together. Hypermesh offers advanced automation capabilities that enable the optimization of meshes based on specific quality standards, modification of preexisting meshes using morphing techniques, and creation of mid-surfaces from models with different thicknesses. The FEM, also known as meshed models, was comprised of 3D tetrahedral elements with four nodes each. A GM consists solely of lines, surfaces, and volumes. A FEM consists of nodes and elements (Figure 3).

**Figure 4:**
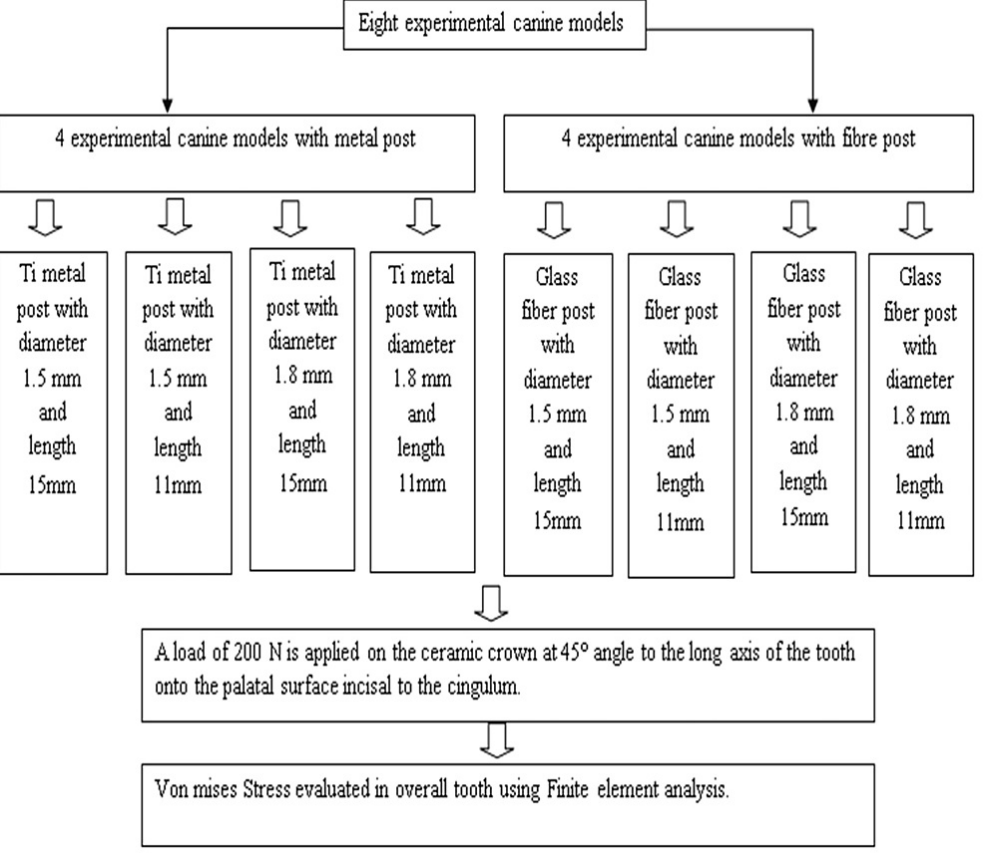
Flow chart of the methodology and grouping Ti, titanium

A total of eight experimental models were developed that portrayed different components of the structure, including the gutta-percha, post, core, root, and crown. The periodontal ligament was shown as a layer with a thickness of 0.3 mm surrounding the surface of the root. The cement layer between the post and the dentin was insufficiently thick to be accurately represented in the finite element simulation. However, the cement was considered a component of the dentin due to the comparable mechanical properties shared by both materials. The lack of a cement layer in the simulation was not anticipated to result in any substantial errors. This study contrasted two distinct categories of posts. These are glass fiber posts and Ti metal posts. The study examined two post diameter architectures: 1.5 mm and 1.8 mm. The study evaluated two post length configurations: one with a length of 15 mm (12 mm in the root, 3 mm coronal, and 4 mm remaining gutta-percha) and another with a length of 11 mm (8 mm in the root, 3 mm coronal, and 8 mm remaining gutta-percha). The models were categorized and further organized into subgroups as outlined below: Models 1 and 5 were constructed using Ti and glass fiber posts, with a diameter of 1.5 mm and a length of 15 mm. They also had a composite core and an all-ceramic crown. Models 2 and 6 were similar, but with a length of 11 mm. Models 3 and 7 had a larger diameter of 1.8 mm while still maintaining a length of 15 mm. Models 4 and 8 had the same diameter but a shorter length of 11 mm (Figure 4).

Determining the characteristics of the material

The elements have been allocated with values for modulus of elasticity and Poisson’s ratio. Acquiring accurate parameters can be a challenging endeavor, requiring considerable estimation when there is limited information available from testing materials or literature. The FEM required accurate assignment of material attributes to accurately simulate the behavior of the material under study. By entering a certain set of material parameters into the finite element program, one can easily receive a corresponding set of numerical outputs. The position, size, and trajectory of force delivery can be readily adjusted to replicate clinical scenarios. The study was based on a few specific suppositions. The assumption was made that all the materials in the model were homogeneous and isotropic. The cement layer was considered to be a component of the dentin due to the same mechanical attributes of dentin and cement.

Establishing the boundary characteristics

The models were meshed using first-order tetrahedral elements, with each node having three degrees of freedom. This led to a total of 67,278 nodes and 357,333 elements, each around 0.3 mm in dimension. The outermost nodes of the alveolar bone were immobilized in all orientations constituting the boundary conditions.

Force application

A 200 N load was exerted on the ceramic crown at a 45° inclination relative to the long axis of the tooth, namely on the palatal surface above the cingulum. The occlusal load of typical magnitude was only taken into account when static loading was given to the tooth. It was divided into vertical (y-axis) and horizontal (x-axis) components. The von Mises stress was used to compute the maximum stresses in the tooth structure and post.

Implementation of the analysis and subsequent interpretation of the findings

The ANSYS software Version 12.1 (Ansys, Inc., Canonsburg, Pennsylvania, United States) was utilized to conduct a linear static analysis, and interpretations of the applied load characteristics were made. ANSYS is a popular FEA code utilized in computer-aided engineering. It enables the creation of computer models for structures, machinery parts, or systems. These models can be subjected to operational loads and standards of design, allowing for the examination of physical responses such as stress values, temperature distributions, pressure, and displacement. The failure was determined by the correlation between a larger von Mises stress value and an increased likelihood of failure. The resulting stresses were then contrasted with the ultimate tensile strength of the material [9]. In a similar vein, structures were noted to shift when forces were surpassed. The pressures and displacements were visualized through a range of different colors. The color red indicates the highest level of stress, whereas dark blue represents the lowest level of stress. von Mises stress is the criterion employed to assess the structure from a stress perspective. The study involved stress analysis, which was conducted employing the equivalent von Mises stress measured in megapascal (MPa).

## Results

The deflection of the teeth appeared nearly the same in all four situations. However, the deflection was significantly greater for a lengthier post. Based on the simulation findings, the greatest deflection of approximately 0.126 mm was seen at the crown region for the given loads being applied and boundary parameters. The FEM of von Mises stress in the crown revealed that the load application area experiences the most stress. The FEM of von Mises stress in the core revealed that the highest stress was located on the palatal side at the center. The FEA of von Mises stress in the Ti post revealed that the highest stress was found in the upper section. The FEA of von Mises stress in the periodontal ligament revealed that the highest stress was found in the cervical region. The FEA of von Mises stress in the cortical bone indicated that the highest stress in the cortical bone was detected near the fixation location, and it remained constant in all cases.

The deflection of the teeth remained nearly uniform in all four situations, except when a longer post was used, where it exhibited a significantly greater deflection. Based on the simulation findings, the highest deflection was found at the crown part, with a value of approximately 0.126 mm, under the given loads being applied and boundary parameters. The FEA of von Mises stress in the crown demonstrated that the load application zone experiences the highest level of stress. The FEA of von Mises stress in the core reveals that the highest stress was seen on the palatal side at the center. The FEA of von Mises stress in the fiber post revealed that the highest stress was seen in the upper section, and that of dentine was revealed to be seen in specific areas. The FEA of von Mises stress in the periodontal ligament revealed that the highest stress occurred in the cervical region. The FEA of von Mises stress in the spongy bone indicated that the highest level of stress was found in the apical area. Table 1 presents the von Mises stress distribution in various sections of Ti and fiber posts.

**Table 1:** von Mises stress in different parts of Ti and fiber post Ti, titanium

Part	Ti post				Fiber post			
	Model 1	Model 2	Model 3	Model 4	Model 5	Model 6	Model 7	Model 8
Composite core	49.5	47.5	49.1	48.4	48.8	46.9	48.3	47.5
Post	43.3	42.6	38.9	37.4	28.1	27.5	26.2	25.8
Dentine	13	13.1	12.8	12.8	13.6	13.5	13.6	13.6
Ceramic crown	200.5	200.4	200.5	200.4	200.4	200.3	200.4	200.3
Periodontal ligament	3.2	3.2	3.2	3.2	3.2	3.2	3.2	3.2
Spongy bone	3.8	3.7	3.8	3.7	3.7	3.7	3.7	3.7
Cortical bone	31.7	31.7	31.7	31.7	31.7	31.7	31.7	31.7

**Figure 5:**
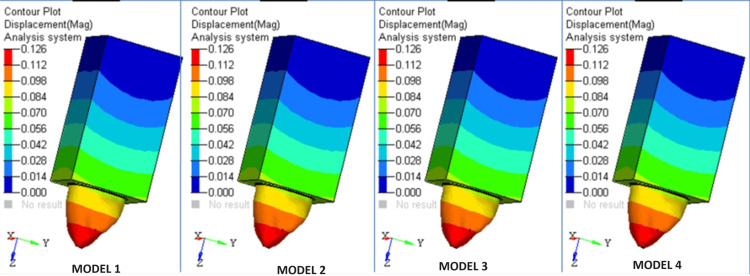
Heat map of the different models

**Figure 6:**
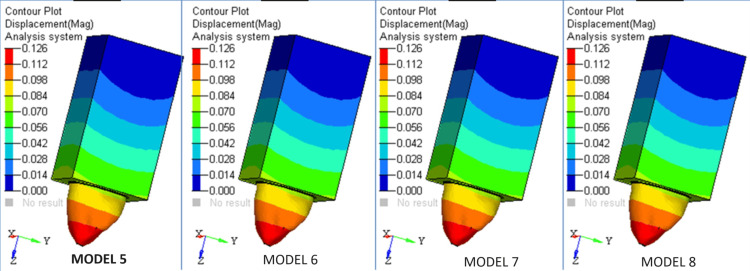
Heat map of the Models used

The highest level of stress in the composite core was recorded in Model 1 (Figures 5,6).

The stress in the core with the Ti post was greater than that in the core with the fiber post. A core reinforced with a fiber post measuring 1.5 mm in diameter and 11 mm in length exhibited lower levels of stress in comparison to other scenarios. Therefore, the fiber post is superior in comparison to the Ti post. In contrast to the stress fluctuations in the composite core of each Ti and fiber post model, the highest level of stress carried through the dentin can be detected in cases using fiber posts as opposed to Ti posts. The transmission of stresses to dentine was limited when using a Ti post with a diameter of 1.8 mm. There was no noticeable change in stress levels when the length of the post was altered.

The design with a diameter of 1.5 mm and a length of 15 mm showed the highest level of stress in the Ti post. The lowest possible stress was seen when the diameter was 1.8 mm and the length was 11 mm. The fiber post design with a diameter of 1.5 mm and a length of 15 mm exhibited the highest level of stress. The lowest level of tension was seen when the diameter was 1.8 mm and the length was 11 mm. The stress levels in the Ti post were greater than those in the fiber post. However, the difference in post material stress fluctuation for various lengths was not statistically significant.

## Discussion

Only a limited number of studies have examined the impact of both post diameter and length on how stresses are distributed in a maxillary canine tooth. Thus, this experiment was conducted to analyze the pattern of distribution of stress in a tooth that was restored using metal and fiber posts with different diameters (1.5 and 1.8 mm) and lengths (11 mm and 15 mm) using FEA. A parallel post was used for this study due to its uniform size and diameter across all examined lengths. The width of the canal opening of the maxillary canine, as reported in previous research [11,12], ranges from 1.47 to 1.78 mm. Therefore, the diameter of the posts in this investigation was chosen to be 1.5 mm and 1.8 mm.

FEA is the preferred method for obtaining an optically realistic investigation that includes comprehensive dental anatomy and a computational approach. In this study, the periodontal ligament and alveolar bone were modeled, emulating clinical conditions. The periodontal ligament has a lower modulus of elasticity, which permits the tooth to reach its lowest point and facilitates its motion. Due to its similar physical qualities to dentin, cementum, which is a thin layer, was deemed superfluous to be classified as a distinct layer from dentin. By employing FEA, it is possible to correctly analyze the stress caused by evaluating the areas of stress concentration. It is more efficient as it avoids the time spent on standardization concerns or on processing several samples, as is the case with mechanical tests. Due to these benefits, this approach has been employed to examine the mechanical properties of teeth that have had endodontic treatment and have been exposed to various procedures and restorations [9].

In 1972, Helfer et al. contended that the loss of water (10%) in teeth without pulp could have an impact on their characteristics [13]. Nevertheless, research juxtaposing certain characteristics, such as microhardness, modulus of elasticity, and tensile strengths, in teeth with vital pulp and teeth without pulp observed that these characteristics had minimal impact on the ability of the teeth to resist fractures, despite observing some alterations in relative humidity and characteristics [14-16]. Due to structural alterations in the dentin that resulted in a reduction of water and collagen cross-linking following root canal therapy, endodontically treated teeth were once thought to be more susceptible to fracture [17]. It is well known that elevated cuspal deflection during function is an indication of the decline of structural integrity related to access preparation, and this increases the risk of fractures. The presence of anterior and canine guidance enables the prompt disengagement of molars and premolars during lateral or protrusive actions, such as mastication [18]. During laterotrusion, the tip of the lower canine contacts the mesial groove of the upper canine [19]. However, much research has not been noticed in the literature about stress distribution in canine teeth that are endodontically treated and reconstructed using post and core. Therefore, this tooth was chosen for the investigation.

Post and core restorations are intricate systems composed of multiple components. The degree of stress distribution within the framework is multiaxial, inconsistent, and influenced by the strength and direction of external forces applied [1]. A tooth that has been rebuilt with a fiber post exhibits a uniform stress distribution, resembling that of a natural tooth. Conversely, the metal post approach caused focused pressures that impacted both the connection between the post and cement, and this, in turn, resulted in tooth fracture [20]. Ti in its pure form exhibits excellent biocompatibility, corrosion resistance, and low heat conductivity. However, it is much less stiff than stainless steel [21]. In their study, Mitsui et al. [22] examined the fracture durability of bovine teeth that were replaced using five distinct intraradicular post frameworks: cast metal posts, Ti posts, glass fiber posts, carbon fiber posts, and zirconium posts. The Ti posts had a greater average fracture resistance score in comparison to glass fiber and zirconium posts and demonstrated comparable results to carbon fiber posts. The conclusion drawn by the authors is that Ti and carbon fiber posts are the most appropriate choices. This study contradicts our findings.

Multiple contributors provide suggestions regarding the length of posts. According to a review article by Goodacre and Spolnik [23], the post length should ideally be three-quarters of the total length of the root canal, or at the very least, the length of the crown. It is advised that a remaining 4-5 mm of gutta-percha should be present at the apex to ensure a sufficient seal. Sorensen and Martinoff [24] found a 97% success rate in a retrospective investigation when the length of the post was equal to or greater than the height of the crown. As per the findings of Neagley, a post must have a minimum length of 8 mm [8]. Research has demonstrated that forces tend to accumulate at the highest point of the bone during the process of chewing. For teeth that have metal posts, forces likewise tend to be concentrated near the tip of the post. Thus, it is essential for a post to consistently protrude apically beyond the crest of the bone [25]. A recent study conducted by Abramovitz et al. [26] has shown that a 3-mm layer of gutta-percha does not reliably seal the apex. As a result, it is suggested to use a layer of 4-5 mm instead. Based on these factors, the post lengths of 11 mm and 15 mm were chosen for this study. To maximize the benefits of increased bonding surface between the post and tooth structure, and additionally, between the post and core material, it is advisable to utilize larger sizes of fiber posts. By employing this method, it is possible to enhance the cohesion and durability of the central material [7].

Irrespective of the content of the post, when the total length of the post increased, the overall displacement decreased [27]. Our findings align with those of Cailleteau et al. [28], who observed a modification in the flexure resistance of the tooth structure when the post was introduced into the root canal. The stress distribution shifted from the coronal region to the apical region of the root. The magnitude of the tension was contingent upon the length of the post. The fiber post group exhibited a greater overall displacement compared to the metal post groups. This could be attributed to the fiber post having a significantly lower elastic modulus compared to the metal post. Consequently, the group using fiber posts exhibited more elasticity and a bigger overall displacement.

Additionally, the direction in which force was applied to the canine teeth was derived from earlier research [20,29]. Model 1 exhibited the highest level of stress in the composite core, as found in this investigation. The stress in the core with the Ti post was greater than that in the core with the fiber post. A core placed with fiber posts measuring 1.5 mm in diameter and 11 mm in length exhibits lower levels of stress when viewed alongside alternative scenarios. The fiber post exhibited superior performance in comparison to the Ti post. Furthermore, the relationship between the low elasticity modulus of the glass fiber post and ceramic, as well as the bonding between ceramic, composite resin core, and resin cement, led to the selection of all-ceramic crowns for this investigation. By utilizing a fiber post, these characteristics produce a flexible, sophisticated restorative system with mechanical qualities comparable to healthy teeth.

According to this study, the stress transferred to dentin was negligible when using a Ti post with a diameter of 1.8 mm. This could be because the modulus of elasticity of the fiber post is similar to that of dentin, which is approximately 20 GPa. Additionally, the fiber post is five to 10 times more flexible than high-modulus metal posts. This flexibility enables the post to effectively absorb stress and avert root fractures. The fiber post exhibits superior stress distribution to dentin compared to the metal post. Although Ti posts induced stress concentration within the post itself, they also increased the likelihood of root breakage. Metal posts, because of their inflexibility and rigidity characteristics, transmit forces in a linear direction, exerting a wedging impact on the tooth structure, akin to a metal wedge on a wooden object [3]. There was no substantial fluctuation in stress seen in dentin when altering the length of the post, but it was relatively lower for shorter posts. The investigation, done by Pegoretti et al., found that glass fiber posts used to replace teeth resulted in a lower concentration of stress within the root compared to metal and carbon fiber posts. When there is a significant variation in Young’s modulus between the dentin and the posts, the stress distribution on the tooth surface becomes less uniform, resulting in the formation of stress concentration zones on the dentin [30].

The FEA is a precise numerical technique used in stress analysis, specifically in dental biomechanics [9]. It allows for the determination of an approximate overall solution to the original problem [10]. A set of concurrent formulas is generated and resolved to determine the expected stress distributions in each component across a structure. The variables can be controlled with the utmost accuracy using computer technology, thereby removing any variance caused by sampling errors. To carry out this FEM analysis, the values for Poisson’s ratio and Young’s modulus of elasticity for all components were obtained from earlier research [1,9]. Performing FEM analysis many times will consistently produce identical results with 100% certainty. Hence, it is unequivocal that the outcomes are consistently influenced by modifying the variables rather than by random occurrences. Hence, traditional inferential statistical analysis is typically excluded from FEA [9].

Currently, some constraints must be acknowledged with respect to the current investigation. It was believed that the structures and materials employed in this investigation were homogeneous, isotropic, and linearly elastic. Consequently, the computational modeling differed from the genuine tooth frame and its supporting tissues [9]. Root fractures often occur owing to dynamic stress, which can culminate in a fatigue cycle in clinical situations distinct from static loading. Additional research is necessary to simulate the susceptibility to fracture and the effects of thermal and mechanical cycle loads to precisely predict the stress distribution in teeth that have undergone endodontic treatment. The research should also consider the impact of various restorative materials on the strength of the teeth, whether they are compromised or not.

## Conclusions

The composite core exhibited the highest level of stress on Ti posts measuring 1.5 mm in diameter and 15 mm in length. Fiber posts with a diameter of 1.8 mm and a length of 11 mm exhibited lower levels of stress in the cervical region in comparison to Ti posts. Maximum strains happened in the tooth’s cervical region regardless of the materials employed. There was no noticeable alteration in stress when the length and diameter of the post were modified. The highest level of stress absorbed by dentin was detected in cases where a fiber post was used, as opposed to cases where a Ti post was used. The fiber post showed minimal observed stresses; however, stresses transferred to the dentin were greater, which were distributed equally in comparison with Ti post cases. To alleviate stress in the remaining radicular tooth with a significant coronal defect, it is advisable to use a composite resin core along with a fiber post that has a wide diameter and shortened length while staying within the therapeutic stipulations.
